# Supplementary material for: Co-targeting Metabolic Neighbours Constraints Bacterial Adaptive Evolution
Source: bioRxiv. 2026 Jun 6:2026.06.04.729935. Preprint. [Version 1] doi: 10.64898/2026.06.04.729935 (PMC13251999; doi:10.64898/2026.06.04.729935)
Supplement: 1 [file NIHPP2026.06.04.729935v1-supplement-1.pdf]

bioRxiv preprint doi: https://doi.org/10.64898/2026.06.04.729935; this version posted June 6, 2026. The copyright holder for this preprint (which was not certified by peer review) is the author/funder, who has granted bioRxiv a license to display the preprint in perpetuity. It is made available under a CC-BY 4.0 International license.

Supplementary Information

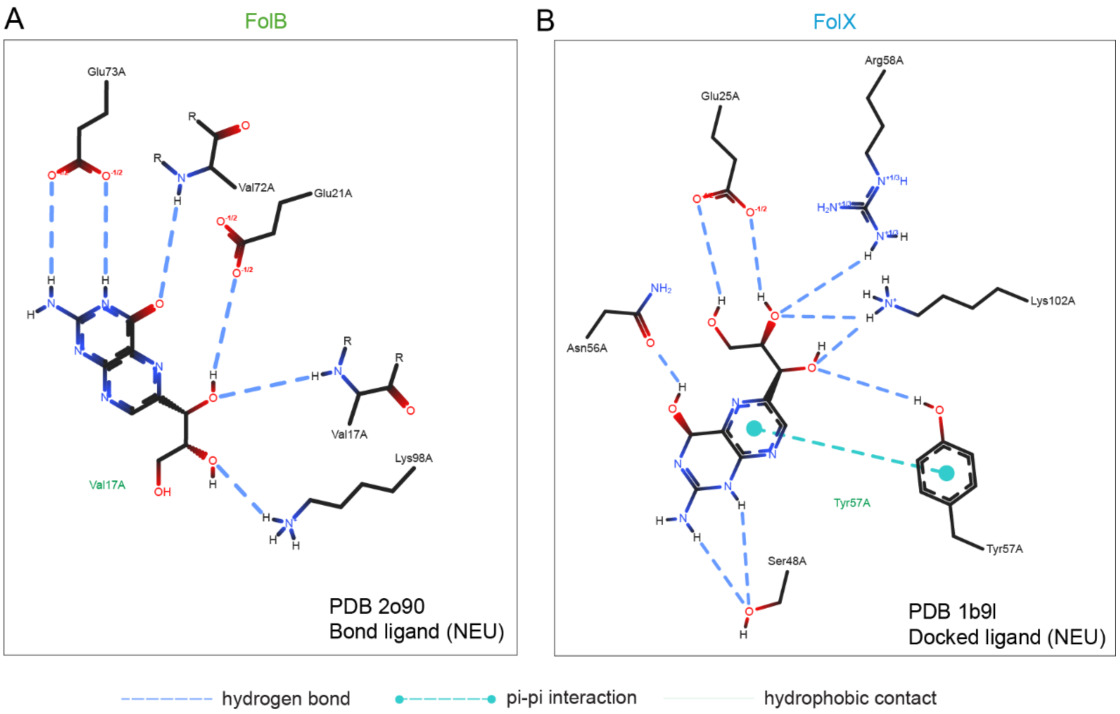

Supplementary Figure 1. **Structural basis of substrate recognition in FolB and FolX.** (A) Ligand interaction map of FolB (PDB: 2O90) bound to its native substrate (NEU). The binding pocket is defined by a network of hydrogen bonds involving residues such as Glu73, Val72, Glu21, and Lys98, which stabilize the pterin scaffold and orient the substrate for catalysis. (B) Ligand interaction map of FolX (PDB: 1B9I) with docked NEU. The binding pocket displays a distinct yet partially overlapping interaction network involving residues such as Glu25, Arg58, Lys102, Asn56, Ser48, and Tyr57. In addition to hydrogen bonding, π–π interactions and hydrophobic contacts contribute to ligand stabilization. Dashed lines indicate hydrogen bonds, dotted lines indicate π–π interactions, and grey arcs represent hydrophobic contacts. Together, these interaction patterns highlight conserved physicochemical features of the binding pockets despite sequence divergence.

bioRxiv preprint doi: https://doi.org/10.64898/2026.06.04.729935; this version posted June 6, 2026. The copyright holder for this preprint (which was not certified by peer review) is the author/funder, who has granted bioRxiv a license to display the preprint in perpetuity. It is made available under a CC-BY 4.0 International license.

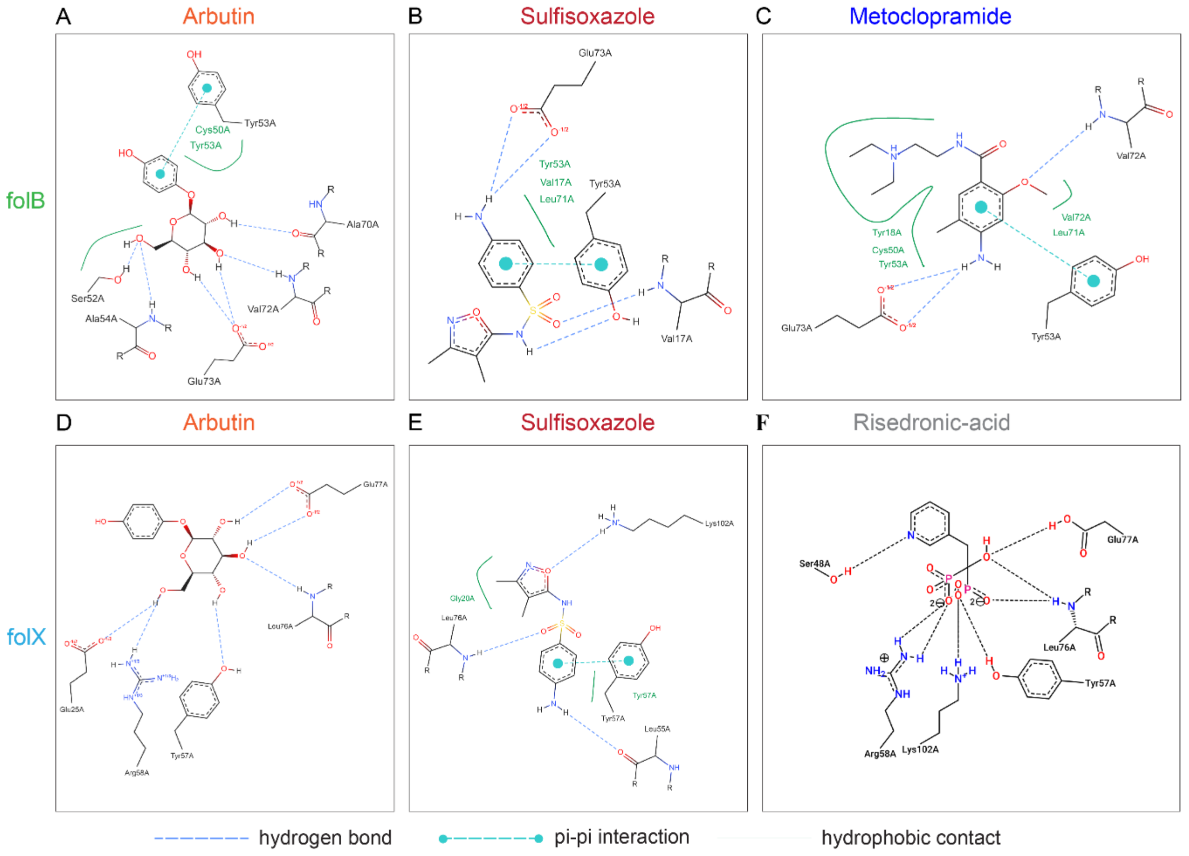

Supplementary Figure 2. **Interaction profiles of representative inhibitors in FolB and FolX binding sites.** (A–C) Interaction maps of representative compounds docked in the FolB binding pocket: Arbutin (A), Sulfisoxazole (B), and Metoclopramide (C). These compounds engage conserved residues through combinations of hydrogen bonding and hydrophobic interactions, with aromatic groups positioned to interact with Tyr53 and surrounding hydrophobic residues. (D–F) Interaction maps of representative compounds docked in the FolX binding pocket: Arbutin (D), Sulfisoxazole (E), and Risedronic acid (F). Binding involves a combination of polar contacts with residues such as Glu25, Arg58, and Lys102, along with aromatic stacking interactions near Tyr57, reflecting adaptation to the FolX pocket architecture. Hydrogen bonds are shown as dashed blue lines, π–π interactions as cyan connectors, and hydrophobic contacts as green arcs. These interaction patterns illustrate how chemically diverse compounds achieve binding through conserved interaction hotspots across both enzymes.

bioRxiv preprint doi: https://doi.org/10.64898/2026.06.04.729935; this version posted June 6, 2026. The copyright holder for this preprint (which was not certified by peer review) is the author/funder, who has granted bioRxiv a license to display the preprint in perpetuity. It is made available under a CC-BY 4.0 International license.

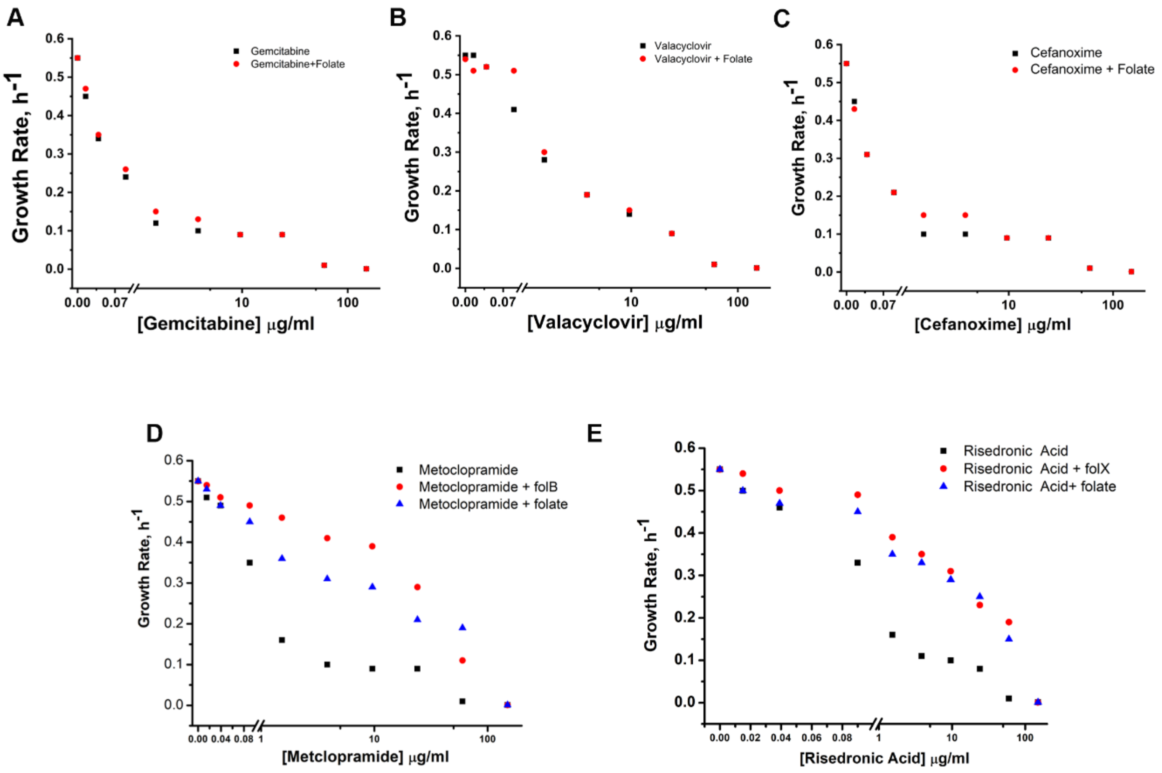

Supplementary Figure 3. Growth rates of bacteria treated with increasing concentrations of (B) Arbutin, (A) Gemcitabine, (B) Valacyclovir, (C) Cefanoxime (D) Metoclopramide, and (E) Risedronic acid measured with (red) and without (black) exogenous folate. Folate restores growth in (D) Metoclopramide, and (E) Risedronic acid treated cells, while no rescue pattern was observed for other inhibitors. Growth rates under Metoclopramide and Risedronic acid exposure in strains overexpressing folB and folX showed rescue trends

bioRxiv preprint doi: https://doi.org/10.64898/2026.06.04.729935; this version posted June 6, 2026. The copyright holder for this preprint (which was not certified by peer review) is the author/funder, who has granted bioRxiv a license to display the preprint in perpetuity. It is made available under a CC-BY 4.0 International license.

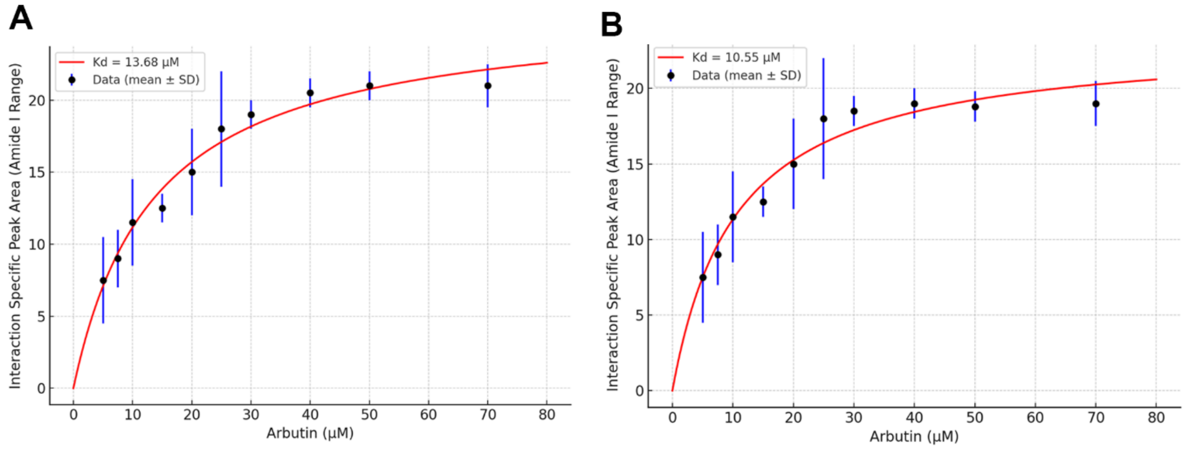

Supplementary Figure 4. **Raman spectroscopy based binding experiments show arbutin co-targets folB and folX.** (A-B) Binding interaction profiles of arbutin with folB and folX. Hyperbolic binding fits show concentration-dependent interaction-specific peak area (amide-I range) for (A) folB and (B) folX, with extracted dissociation constants indicating measurable association between arbutin and each target. Data points represent mean ± SD. We normalized and scaled Raman intensities (including log transformation where appropriate) to enable comparison across proteins and conditions. Because the signal reflects ligand-induced structural perturbations, the derived K_d values represent effective interaction parameters rather than direct thermodynamic binding affinities and may differ from values obtained using thermodynamic methods (e.g., ITC).

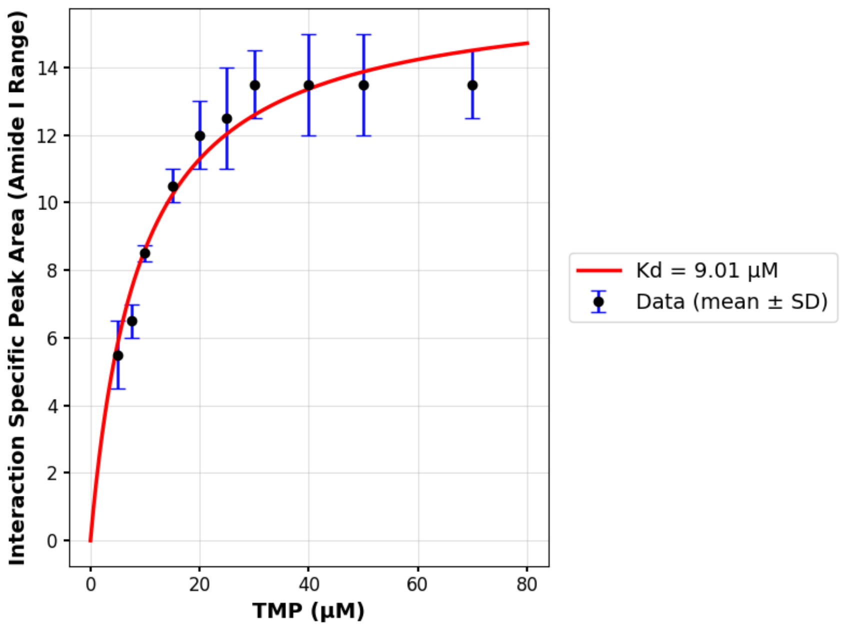

bioRxiv preprint doi: https://doi.org/10.64898/2026.06.04.729935; this version posted June 6, 2026. The copyright holder for this preprint (which was not certified by peer review) is the author/funder, who has granted bioRxiv a license to display the preprint in perpetuity. It is made available under a CC-BY 4.0 International license.

Supplementary Figure 5. **Binding interaction profiles of TMP (Trimethoprim) with folA (DHFR).** Hyperbolic binding fits show concentration-dependent interaction-specific peak area (amide-I range). Data points represent mean ± SD.

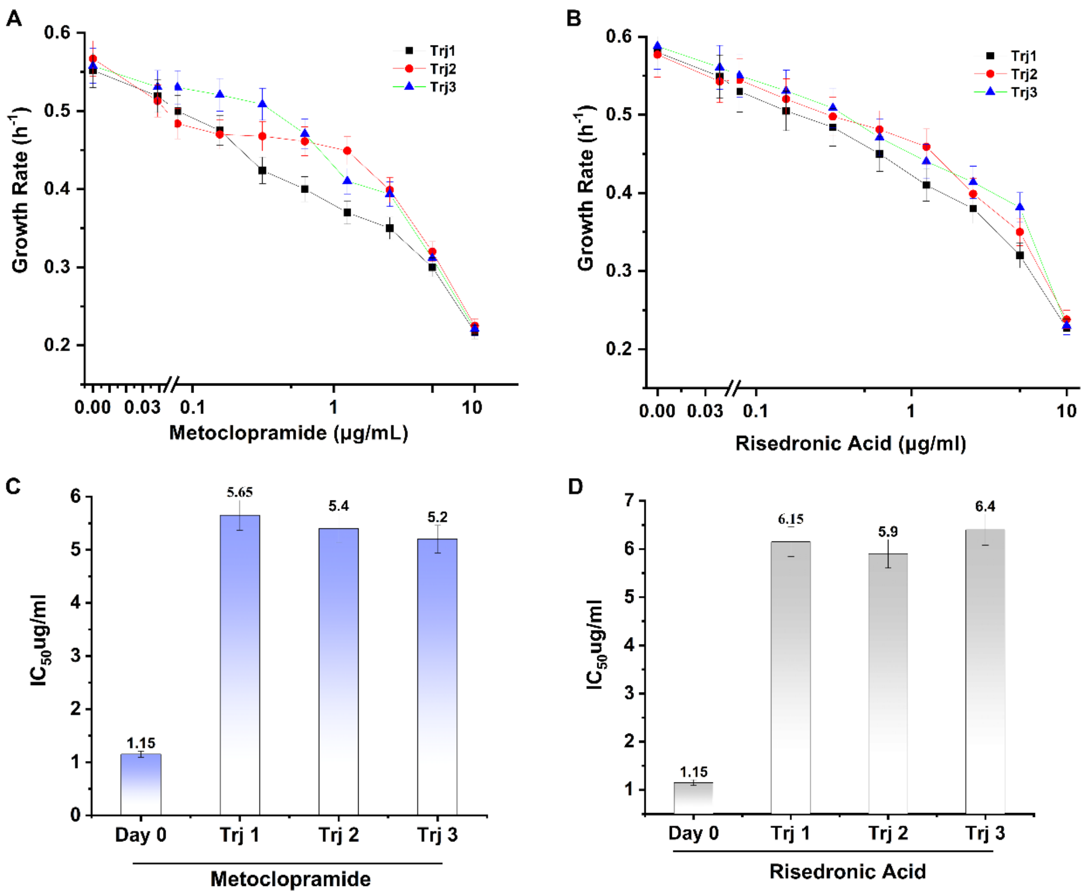

Supplementary Figure 6. **Evolution of resistance under single-target inhibition of FolB and FolX.** (A) Growth-rate measurements for populations evolved under Metoclopramide (FolB inhibitor). Three independent trajectories show reduced growth rates with increasing Metoclopramide concentration, while maintaining higher growth at intermediate concentrations relative to the ancestral strain. Points represent mean values; error bars indicate standard deviation. (B) Growth-rate measurements for populations evolved under Risedronic acid (FolX inhibitor). Three independent trajectories display a progressive reduction in growth rate with increasing drug concentration. Points represent mean values; error bars indicate standard deviation. (C) IC_{50} values for Metoclopramide-evolved populations. The ancestral IC_{50} (1.15 µg/mL) is shown on the left. Evolved populations exhibit elevated IC_{50} values ranging from ~5.2 to 5.7 µg/mL across independent trajectories. (D) IC_{50} values for Risedronic acid–evolved populations. The ancestral IC_{50} (1.15 µg/mL) is shown on the left. Evolved populations show increased IC_{50} values ranging from ~5.9 to 6.4 µg/mL across independent trajectories.

bioRxiv preprint doi: https://doi.org/10.64898/2026.06.04.729935; this version posted June 6, 2026. The copyright holder for this preprint (which was not certified by peer review) is the author/funder, who has granted bioRxiv a license to display the preprint in perpetuity. It is made available under a CC-BY 4.0 International license.

Supplementary Table 1. 19 tested drugs and their IC_{50} from growth measurements.

**No.**	**Name**	**Predicted target**	**IC_{50} (μg/ml)**
1	Valganciclovir	Both folB and folX	1.9
2	Arbutin	Both folB and folX	1.3
3	Sulfisoxazole	Both folB and folX	0.25
4	Nitenpyram	Both folB and folX	2.5
5	Cytarabine	Both folB and folX	N/A
6	Droxidopa	Both folB and folX	N/A
7	Vidarabine	Both folB and folX	N/A
8	Gemcitabine	folB	0.35
9	Valaciclovir	folB	0.9
10	Cefmenoxime	folB	0.2
11	Amiloride	folB	N/A
12	Lodoxamide	folB	N/A
13	Metoclopramide	folB	N/A
14	Ribavirin	folB	N/A
15	Zanamivir	folB	N/A
16	Aztreonam	folX	1.2
17	Nelarabine	folX	N/A
18	Nitazoxanide	folX	N/A
19	Risedronic Acid	folX	N/A

bioRxiv preprint doi: https://doi.org/10.64898/2026.06.04.729935; this version posted June 6, 2026. The copyright holder for this preprint (which was not certified by peer review) is the author/funder, who has granted bioRxiv a license to display the preprint in perpetuity. It is made available under a CC-BY 4.0 International license.

Supplementary Table 2. Functional classification of mutations in the Arbutin-evolved trajectory Trj1.

Start	End	Variant Type	Gene Name	CDS	Gene_type
3825094	3825095	snp	ispD	4-diphosphocytidyl-2C-methyl-D-erythritol synthase	Metabolic
3808101	3808102	snp	pphB	serine/threonine-specific protein phosphatase 2	Others
3620491	3620491	deletion	nadB	quinolinate synthase, L-aspartate oxidase (B protein) subunit	Metabolic
3513759	3513760	snp	yfgF	cyclic-di-GMP phosphodiesterase, anaerobic	Regulatory
3507298	3508521	insertion	ppk	polyphosphate kinase, component of RNA degradosome	Metabolic
3375626	3375627	snp	yfdX	uncharacterized protein	Others
3174042	3174043	snp	arnA	fused UDP-L-Ara4N formyltransferase/UDP-GlcA C-4'-decarboxylase	Others
2781716	2781897	insertion	yeeA	putative transporter, FUSC family inner membraneprotein	Cell envelope
2579380	2579380	deletion	yobB	C-N hydrolase family protein	Others
2542679	2542680	snp	sdaA	L-serine dehydratase 1	Metabolic
2280353	2281130	insertion	uidA	beta-D-glucuronidase	Metabolic
2226965	2226966	snp	ynfA	UPF0060 family inner membrane protein	Cell envelope
1900802	1900802	deletion	ydaM	diguanylate cyclase, csgD regulator	Regulatory
1710335	1710335	deletion	ychF	putative GTP-binding protein; catalase inhibitor protein	Regulatory
1362767	1362767	deletion	ompF	outer membrane porin 1a (Ia;b;F)	Cell envelope
1039807	1039808	snp	rhsO	pseudogene, Rhs family	Others
773309	773310	snp	ybcF	putative carbonate kinase	Metabolic
459495	459496	snp	yahO	periplasmic protein	Cell envelope
459494	459495	snp	yahO	periplasmic protein	Cell envelope
459493	459494	snp	yahO	periplasmic protein	Cell envelope
323274	323275	insertion	gpt	xanthine phosphoribosyltransferase; xanthine-guanine phosphoribosyltransferase	Metabolic
4419216	4419217	snp	nusA	transcription termination/antitermination L factor	Regulatory
4398233	4398234	snp	yhbT	SCP-2 sterol transfer family protein	Others
4355170	4355171	snp	tdcR	L-threonine dehydratase operon activator protein	Regulatory
4352295	4352296	snp	tdcB	L-threonine dehydratase, catabolic	Metabolic
4220150	4220151	snp	mqsA	antitoxin for MqsR toxin; transcriptional repressor	Efflux and Stress response
4220149	4220149	deletion	mqsA	antitoxin for MqsR toxin; transcriptional repressor	Efflux and Stress response
4041695	4041696	snp	recJ	ssDNA exonuclease, 5' --> 3'-specific	Regulatory

bioRxiv preprint doi: https://doi.org/10.64898/2026.06.04.729935; this version posted June 6, 2026. The copyright holder for this preprint (which was not certified by peer review) is the author/funder, who has granted bioRxiv a license to display the preprint in perpetuity. It is made available under a CC-BY 4.0 International license.

3931049	3931050	snp	recD	exonuclease V (RecBCD complex), alpha chain	Regulatory
3914020	3914021	snp	rlmM	23S rRNA C2498 2'-O-ribose methyltransferase, SAM-dependent	Regulatory
3899860	3899861	snp	sdaB	L-serine dehydratase 2	Metabolic

Supplementary Table 3. Functional classification of mutations in the Arbutin-evolved trajectory Trj2.

Start	End	Variant Type	Gene Name	CDS	Gene_type
3825094	3825095	snp	metZ	Unknown	Others
3808101	3808102	snp	fucP	L-fucose transporter	Others
3620491	3620491	deletion	csiD	carbon starvation protein	Efflux and Stress response
3513759	3513760	snp	yfhH	putative DNA-binding transcriptional regulator	Regulatory
3507298	3508521	insertion	purL	phosphoribosylformyl-glycineamide synthetase	Metabolic
3375626	3375627	snp	acrD	aminoglycoside	Regulatory
3174042	3174043	snp	folC	bifunctional folylpolyglutamate synthase/ dihydrofolate synthase	Folate pathway
2781716	2781897	insertion	wzc	colanic acid production tyrosine-protein kinase;autokinase; Ugd phosphorylase	Metabolic
2579380	2579380	deletion	cheA	sensory histidine kinase/signal sensing protein	Regulatory
2542679	2542680	snp	ruvB	ATP-dependent DNA helicase, component of RuvABC resolvasome	Metabolic
2280353	2281130	insertion	sodB	superoxide dismutase, Fe	Efflux and Stress response
2226965	2226966	snp	uidB	glucuronide transporter	Others
1900802	1900802	deletion	ydbH	putative membrane-anchored protein	Cell envelope
1710335	1710335	deletion	rssB	PcnB-degradosome interaction factor; response regulator	Regulatory
1362767	1362767	deletion	pqiA	paraquat-inducible, SoxRS-regulated inner membrane protein	Cell envelope
1039807	1039808	snp	sdhA	succinate dehydrogenase, flavoprotein subunit	Metabolic

bioRxiv preprint doi: https://doi.org/10.64898/2026.06.04.729935; this version posted June 6, 2026. The copyright holder for this preprint (which was not certified by peer review) is the author/funder, who has granted bioRxiv a license to display the preprint in perpetuity. It is made available under a CC-BY 4.0 International license.

773309	773310	snp	sfmZ	response regulator family protein	Regulatory
459495	459496	snp	prpE	propionate--CoA ligase	Metabolic
459494	459495	snp	prpE	propionate--CoA ligase	Metabolic
459493	459494	snp	prpE	propionate--CoA ligase	Metabolic
323274	323275	insertion	ykfI	CP4-6 prophage; toxin of the YkfI-YafW toxin-antitoxin system	Efflux and Stress response
4419216	4419217	snp	accB	acetyl CoA carboxylase, BCCP subunit	Metabolic
4398233	4398234	snp	aaeA	p-hydroxybenzoic acid efflux system component	Efflux and Stress response
4355170	4355171	snp	gltB	glutamate synthase, large subunit	Metabolic
4352295	4352296	snp	arcB	aerobic respiration control sensor histidine protein kinase, cognate to two-component response regulators ArcA and RssB	Regulatory
4220150	4220151	snp	rlmG	23S rRNA m(2)G1835 methyltransferase, SAM-dependent	Metabolic
4220149	4220149	deletion	rlmG	23S rRNA m(2)G1835 methyltransferase, SAM-dependent	Metabolic
4041695	4041696	snp	pppA	bifunctional prepilin leader peptidase/ methylase	Metabolic
3931049	3931050	snp	uacT	uric acid permease	Others

Supplementary Table 4. Functional classification of mutations in the Arbutin-evolved trajectory Trj3.

**Start**	**End**	**Variant Type**	**Gene Name**	**CDS**	**Gene_type**
3824592	3824593	snp	xdhD	putative hypoxanthine oxidase, molybdopterin-binding/Fe-S binding	Folate pathway
3807918	3807919	snp	hyuA	D-stereospecific phenylhydantoinase	Metabolic
3620308	3620308	deletion	pphB	serine/threonine-specific protein phosphatase 2	Metabolic
3513759	3513760	snp	yfjW	CP4-57 prophage; putative inner membrane protein	Recombination
3507298	3508521	insertion	yfjQ	CP4-57 prophage; uncharacterized protein	Recombination
3375626	3375627	snp	hscB	HscA co-chaperone, J domain-containing protein Hsc56; IscU-specific chaperone HscAB	Others
3174042	3174043	snp	ypdA	sensor kinase regulating yhjX; pyruvate-responsive YpdAB two-component system	Regulatory
2781716	2781897	insertion	metG	methionyl-tRNA synthetase	Metabolic

bioRxiv preprint doi: https://doi.org/10.64898/2026.06.04.729935; this version posted June 6, 2026. The copyright holder for this preprint (which was not certified by peer review) is the author/funder, who has granted bioRxiv a license to display the preprint in perpetuity. It is made available under a CC-BY 4.0 International license.

2579380	2579380	deletion	hchA	glyoxalase III and Hsp31 molecular chaperone	Others
2542679	2542680	snp	amyA	cytoplasmic alpha-amylase	Metabolic
2280353	2281130	insertion	btuC	vitamin B12 ABC transporter permease	Others
2226965	2226966	snp	ydhX	putative 4Fe-4S ferridoxin-type protein; FNR, Nar, NarP-regulated protein	Metabolic
1900802	1900802	deletion	trg	methyl-accepting chemotaxis protein III, ribose and galactose sensor receptor	Regulatory
1710335	1710335	deletion	osmB	osmotically and stress inducible lipoprotein	Efflux and Stress response
1362767	1362767	deletion	torR	response regulator in two-component regulatory system with TorS	Regulatory
1039807	1039808	snp	ybhA	pyridoxal phosphate (PLP) phosphatase	Metabolic
773309	773310	snp	nfrB	bacteriophage N4 receptor, inner membrane subunit	Recombination
459495	459496	snp	lacZ	beta-D-galactosidase	Metabolic
459494	459495	snp	lacZ	beta-D-galactosidase	Metabolic
459493	459494	snp	lacZ	beta-D-galactosidase	Metabolic
323274	323275	insertion	yafZ	CP4-6 prophage; conserved protein	Recombination
4419033	4419034	snp	tsgA	putative transporter	Others
4398050	4398051	snp	yheO	putative PAS domain-containing DNA-binding transcriptional regulator	Regulatory
4354987	4354988	snp	rpoA	RNA polymerase, alpha subunit	Regulatory
4352112	4352113	snp	trkA	NAD-binding component of TrK potassium transporter	Others
4219967	4219968	snp	dacB	D-alanyl-D-alanine carboxypeptidase	Metabolic
4219966	4219966	deletion	dacB	D-alanyl-D-alanine carboxypeptidase	Metabolic
4041512	4041513	snp	glnE	fused deadenylyltransferase/adenylyltransferase for glutamine synthetase	Metabolic
3930866	3930867	snp	speC	ornithine decarboxylase, constitutive	Metabolic

Supplementary Table 5. Gene family-level similarity of mutations across Arbutin-evolved trajectories.

**Gene family (name-based)**	**Trj1 genes**	**Trj2 genes**	**Trj3 genes**	**Similarity Pattern**
rec / recombination	recJ, recD	–	–	**Trj1-specific (repair family hit)**
ruv / recombination	–	ruvB	–	**Trj2-specific (repair family hit)**

bioRxiv preprint doi: https://doi.org/10.64898/2026.06.04.729935; this version posted June 6, 2026. The copyright holder for this preprint (which was not certified by peer review) is the author/funder, who has granted bioRxiv a license to display the preprint in perpetuity. It is made available under a CC-BY 4.0 International license.

DNA repair helicase/exonuclease machinery (rec+ruv as a unit)	recJ, recD	ruvB	–	**Yes (Trj1 ↔ Trj2) at the “recomb/repair gene-family” level**
rlm / rRNA methylation	rlmM	rlmG	–	**Yes (Trj1 ↔ Trj2) (same rlm family, different paralogs)**
sda / serine deaminase	sdaA, sdaB	–	–	**Trj1-specific (clear within-trajectory family hit)**
tdc / threonine/serine catabolism regulon	tdcR, tdcB	–	–	**Trj1-specific (within-trajectory family hit)**
uid / β-glucuronidase operon family	uidA	uidB	–	**Yes (Trj1 ↔ Trj2) (same uid family, different genes)**
yfj*	–	–	yfjW, yfjQ	**Trj3-specific (within-trajectory family hit)**
ygf*	ygfF	ygfK	–	**Yes (Trj1 ↔ Trj2) (same ygf family, different genes)**
met* (methionine-related genes; name family only)	–	metZ	metG	**Weak yes (Trj2 ↔ Trj3) (both “met” family but not same function)**
pphB (exact gene repeat)	pphB	–	pphB	**Strong yes (Trj1 ↔ Trj3) (only clear exact gene overlap)**
Two-component / sensory kinase-regulator family (name-based examples)	–	arcB, cheA	torR	**Yes (Trj2 ↔ Trj3) (same broad “sensor/regulator” gene-family theme, but names differ)**

bioRxiv preprint doi: https://doi.org/10.64898/2026.06.04.729935; this version posted June 6, 2026. The copyright holder for this preprint (which was not certified by peer review) is the author/funder, who has granted bioRxiv a license to display the preprint in perpetuity. It is made available under a CC-BY 4.0 International license.

1146 Supplementary Table 6. Functional classification of mutations in the Risedronic acid-evolved
1147 trajectory Trj1 (folX inhibition).

Start	End	Variant Type	Gene Name	CDS	Gene_type
3808094	3808095	snp	galR	galactose-inducible d-galactose regulon transcriptional repressor; autorepressor	Regulatory
3620484	3620484	deletion	norR	anaerobic nitric oxide reductase DNA-binding transcriptional activator	Regulatory
3513752	3513753	snp	yfiB	OM lipoprotein putative positive effector of YfiN activity	Cell envelope
3507291	3508514	insertion	pheA	chorismate mutase and prephenate dehydratase, P-protein	Metabolic
3375619	3375620	snp	guaB	IMP dehydrogenase	Metabolic
3174041	3174042	snp	tfaS	pseudogene	Others
2781715	2781896	insertion	insE1	IS3 transposase A	Transposable element (TE) and Recombination
2579379	2579379	deletion	yedK	DUF159 family protein	Regulatory
2542678	2542679	snp	uspC	universal stress protein	Efflux and Stress response
2280352	2281129	insertion	lhr	putative ATP-dependent helicase	Regulatory
2226964	2226965	snp	lhr	putative ATP-dependent helicase	Regulatory
1900801	1900801	deletion	ydbA	pseudogene, autotransporter homolog	Others
1710334	1710334	deletion	yciO	putative RNA binding protein	Regulatory
1362766	1362766	deletion	etk	tyrosine-protein kinase, role in O-antigen capsule formation	Metabolic
1039806	1039807	snp	modF	molybdate ABC transporter ATPase	Others
773308	773309	snp	ybdG	mechanosensitive channel protein	Others
459494	459495	snp	lacZ	beta-D-galactosidase	Metabolic
459493	459494	snp	lacZ	beta-D-galactosidase	Metabolic
459492	459493	snp	lacZ	beta-D-galactosidase	Metabolic
323273	323274	insertion	phoE	outer membrane phosphoporin protein E	Cell envelope
67920	67920	deletion	surA	peptidyl-prolyl cis-trans isomerase (PPIase); survival protein	Metabolic
4419209	4419210	snp	gspC	general secretory pathway component	Others

bioRxiv preprint doi: https://doi.org/10.64898/2026.06.04.729935; this version posted June 6, 2026. The copyright holder for this preprint (which was not certified by peer review) is the author/funder, who has granted bioRxiv a license to display the preprint in perpetuity. It is made available under a CC-BY 4.0 International license.

4398226	4398227	snp	zntR	zntA gene transcriptional activator	Regulatory
4355163	4355164	snp	acuI	putative acryloyl-CoA reductase	Efflux and Stress response
4352288	4352289	snp	csrD	targeting factor for csrBC sRNA degradatio	Others
4220143	4220144	snp	yraQ	putative inner membrane permease	Cell envelope
4220142	4220142	deletion	yraQ	putative inner membrane permease	Cell envelope
4041688	4041689	snp	ygiQ	Radical SAM superfamily protein	Metabolic
3931042	3931043	snp	yggD	MtlR family putative transcriptional repressor	Regulatory
3914013	3914014	snp	scpA	methylmalonyl-CoA mutase	Metabolic
3899853	3899854	snp	ubiI	2-octaprenylphenol hydroxylase, FAD-dependent	Metabolic

Supplementary Table 7. Functional classification of mutations in the Risedronic acid-evolved trajectory Trj2 (folX inhibition).

Start	End	Variant Type	Gene Name	CDS	Gene_type
3808101	3808102	snp	argP	transcriptional regulator for arginine transportand DNA replication genes; replication initiationinhibitor	Regulatory
3620491	3620491	deletion	pyrG	CTP synthetase	Metabolic
3513759	3513760	snp	alaS	alanyl-tRNA synthetase	Metabolic
3507298	3508521	insertion	yqaB	fructose-1-P and 6-phosphogluconate phosphatase	Metabolic
3375626	3375627	snp	lepB	leader peptidase (signal peptidase I)	Cell envelope
3174042	3174043	snp	cysM	cysteine synthase B (O-acetylserine sulfhydrolase B)	Metabolic
2781716	2781897	insertion	yohD	DedA family inner membrane protein	Cell envelope
2579380	2579380	deletion	cobT	nicotinate-nucleotide--dimethylbenzimidazole phosphoribosyltransferase	Metabolic
2542679	2542680	snp	yedS	pseudogene, outer membrane protein	Cell envelope

bioRxiv preprint doi: https://doi.org/10.64898/2026.06.04.729935; this version posted June 6, 2026. The copyright holder for this preprint (which was not certified by peer review) is the author/funder, who has granted bioRxiv a license to display the preprint in perpetuity. It is made available under a CC-BY 4.0 International license.

2280353	2281130	insertion	chbC	N,N'-diacetylchitobiose-specific enzyme IIC component of PTS	Metabolic
2226965	2226966	snp	aroD	3-dehydroquinate dehydratase	Metabolic
1900802	1900802	deletion	ydcR	putative DNA-binding transcriptional regulator and putative aminotransferase	Regulatory
1710335	1710335	deletion	puuP	putrescine importer	Others
1362767	1362767	deletion	rutE	putative malonic semialdehyde reductase	Metabolic
1039807	1039808	snp	bioB	biotin synthase	Metabolic
773309	773310	snp	entF	enterobactin synthase multienzyme complex component, ATP-dependent	Metabolic
459495	459496	snp	mhpT	3-hydroxyphenylpropionic transporter	Others
459494	459495	snp	mhpT	3-hydroxyphenylpropionic transporter	Others
459493	459494	snp	mhpT	3-hydroxyphenylpropionic transporter	Others
323274	323275	insertion	insI1	IS30 transposase	Transposable element (TE) and Recombination
4419225	4419226	snp	malP	maltodextrin phosphorylase	Metabolic
4398242	4398243	snp	ompR	response regulator in two-component regulatory system with EnvZ	Regulatory
4355179	4355180	snp	frlC	fructoselysine 3-epimerase	Others
4352304	4352305	snp	frlA	putative fructoselysine transporter	Others
4327466	4327475	insertion	kefG	potassium-efflux system ancillary protein for KefB, glutathione-regulated	Efflux and Stress response
4220150	4220151	snp	yhdP	DUF3971-AsmA2 domains protein	Cell envelope
4220149	4220149	deletion	yhdP	DUF3971-AsmA2 domains protein	Cell envelope
4041695	4041696	snp	ygjV	Imp-YgjV family inner membrane protein	Cell envelope
3931049	3931050	snp	yqhC	transcriptional activator of yqhD	Regulatory
3914020	3914021	snp	hybC	hydrogenase 2, large subunit	Metabolic
3899860	3899861	snp	insH1	IS5 transposase and trans-activator	Regulatory

1152

bioRxiv preprint doi: https://doi.org/10.64898/2026.06.04.729935; this version posted June 6, 2026. The copyright holder for this preprint (which was not certified by peer review) is the author/funder, who has granted bioRxiv a license to display the preprint in perpetuity. It is made available under a CC-BY 4.0 International license.

Supplementary Table 8. Functional classification of mutations in the Risedronic acid-evolved trajectory Trj3 (folX inhibition).

Start	End	Variant Type	Gene Name	CDS	Gene_type
3490426	3490427	snp	yfhM	bacterial alpha2-macroglobulin colonization factor ECAM; anti-host protease defense factor	Efflux and Stress response
3678037	3678037	deletion	nrdI	NrdEF cluster assembly flavodoxin	Metabolic
3784768	3784769	snp	casD	CRISP RNA (crRNA) containing Cascade antiviral complex protein	Regulatory
3790007	3791230	insertion	ygcB	Cascade complex anti-viral R-loop helicase-annealase Cas3	Regulatory
3922901	3922902	snp	yqeH	putative LuxR family transcriptional regulator	Regulatory
4124485	4124486	snp	hybD	maturation protease for hydrogenase 2	Metabolic
4516631	4516812	insertion	rpsK	30S ribosomal subunit protein S11	Regulatory
89892	89892	deletion	araA	L-arabinose isomerase	Metabolic
126592	126593	snp	murD	UDP-N-acetylmuramoyl-L-alanine:D-glutamate ligase	Metabolic
388142	388919	insertion	paoD	moco insertion factor for PaoABC aldehyde oxidoreductase	Others
442306	442307	snp	yahI	carbamate kinase-like protein	Metabolic
768470	768470	deletion	nfrA	bacteriophage N4 receptor, outer membrane subunit	Cell envelope
958937	958937	deletion	pgm	phosphoglucomutase	Metabolic
1306505	1306505	deletion	mukB	chromosome condensin MukBEF, ATPase and DNA-binding subunit	Others
1629464	1629465	snp	minD	inhibitor of FtsZ ring polymerization; chromosome-membrane tethering protein	Cell envelope
1895962	1895963	snp	ttcC	pseudogene, prophage Rac integration site ttcA duplication;Phage or Prophage Related	Transposable element (TE) and Recombination
2209776	2209777	snp	pntA	pyridine nucleotide transhydrogenase, alpha subunit	Metabolic
2209777	2209778	snp	pntA	pyridine nucleotide transhydrogenase, alpha subunit	Metabolic

bioRxiv preprint doi: https://doi.org/10.64898/2026.06.04.729935; this version posted June 6, 2026. The copyright holder for this preprint (which was not certified by peer review) is the author/funder, who has granted bioRxiv a license to display the preprint in perpetuity. It is made available under a CC-BY 4.0 International license.

2209778	2209779	snp	pntA	pyridine nucleotide transhydrogenase, alpha subunit	Metabolic
2345997	2345998	insertion	ydiP	putative DNA-binding transcriptional regulator	Regulatory
2601351	2601351	deletion	cheA	fused chemotactic sensory histidine kinase in two-component regulatory system	Regulatory
2879311	2879312	snp	yehB	putative outer membrane protein	Cell envelope
2900294	2900295	snp	yehM	uncharacterized protein	Others
2943357	2943358	snp	mglA	methyl-galactoside ABC transporter ATPase	Others
2946232	2946233	snp	mglB	methyl-galactoside transporter subunit	Others
3078377	3078378	snp	gyrA	DNA gyrase (type II topoisomerase), subunit A	Metabolic
3078379	3078379	deletion	gyrA	DNA gyrase (type II topoisomerase), subunit A	Metabolic
3256832	3256833	snp	yfdK	CPS-53 (KpLE1) prophage; conserved protein	Transposable element (TE) and Recombination
3367478	3367479	snp	intZ	CPZ-55 prophage; putative phage integrase	Transposable element (TE) and Recombination
3384507	3384508	snp	eutD	phosphate acetyltransferase	Metabolic
3398667	3398668	snp	nudK	GDP-mannose pyrophosphatase	Others

Supplementary Table 9. Gene family-level similarity of mutations across Risedronic acid-evolved trajectories

Gene-family (name-based)	Trj1 genes	Trj2 genes	Trj3 genes	Similarity pattern
ins* (insertion sequence / IS element genes)	insE1	insI1, insH1	–	Shared Trj1 ↔ Trj2 (TE/IS family repeatedly hit)
yed*	yedK	yedS	–	Shared Trj1 ↔ Trj2 (same yed-family, different members)
mgl*	–	–	mglA, mglB	Trj3-specific within-trajectory family hit

bioRxiv preprint doi: https://doi.org/10.64898/2026.06.04.729935; this version posted June 6, 2026. The copyright holder for this preprint (which was not certified by peer review) is the author/funder, who has granted bioRxiv a license to display the preprint in perpetuity. It is made available under a CC-BY 4.0 International license.

hyb* (hydrogenase-related genes; name-family)	–	–	hybD, hybC	Trj3-specific within-trajectory family hit
frlA / frlC	–	frlA, frlC	–	Trj2-specific within-trajectory family hit
lacZ (exact gene)	lacZ	–	–	Trj1-specific
regulator "-R" family (name pattern only)	galR, norR, zntR	–	–	Trj1 enriched in "R" regulators (pattern, not paralogs)
response regulator / two-component name-family	–	ompR	–	Trj2-specific
chemotaxis "che" family	–	–	cheA	Trj3-specific
DNA gyrase family	–	–	gyrA	Trj3-specific
cell division / partition family (min/muk)	–	–	minD, mukB	Trj3-specific (two separate families but same theme)
nucleotide/redox "nud/nrd" families	–	–	nudK, nrdI	Trj3-specific

Supplementary Table 10. Functional classification of mutations in the Metoclopramide-evolved trajectory Trj1 (folB inhibition).

Start	End	Variant Type	Name	CDS	Gene_type
3490427	3490428	snp	hisS	histidyl tRNA synthetase	Metabolic
3678038	3678038	deletion	csiD	carbon starvation protein	Others
3784769	3784770	snp	ygbN	putative transport protein	Others
3790008	3791231	insertion	cysC	adenosine 5'-phosphosulfate kinase	Metabolic
3922902	3922903	snp	tas	putative NADP(H)-dependent aldo-keto reductase	Metabolic
4124486	4124487	snp	glcB	malate synthase G	Metabolic

bioRxiv preprint doi: https://doi.org/10.64898/2026.06.04.729935; this version posted June 6, 2026. The copyright holder for this preprint (which was not certified by peer review) is the author/funder, who has granted bioRxiv a license to display the preprint in perpetuity. It is made available under a CC-BY 4.0 International license.

4516632	4516813	insertion	acrF	multidrug efflux system protein	Efflux and Stress response
89892	89892	deletion	araB	L-ribulokinase	Metabolic
126592	126593	snp	murD	UDP-N-acetylmuramoyl-L-alanine:D-glutamate ligase	Metabolic
388142	388919	insertion	paoD	moco insertion factor for PaoABC aldehyde oxidoreductase	Metabolic
442306	442307	snp	yahF	putative NAD(P)-binding succinyl-CoA synthase	Metabolic
768470	768470	deletion	aaaD	pseudogene; DLP12 prophage; tail fiber assembly protein family;Phage or Prophage Related	Regulatory
958937	958937	deletion	kdpD	fused sensory histidine kinase in two-component regulatory system with KdpE: signal sensing protein	Regulatory
1306505	1306505	deletion	pncB	nicotinate phosphoribosyltransferase	Metabolic
1629464	1629465	snp	umuC	translesion error-prone DNA polymerase V subunit; DNA polymerase activity	Regulatory
1821893	1821894	insertion	ycjW	LacI family putative transcriptional repressor	Regulatory
1895963	1895964	snp	pfo	pyruvate-flavodoxin oxidoreductase	Metabolic
2209777	2209778	snp	tus	inhibitor of replication at Ter, DNA-binding protein	Regulatory
2209778	2209779	snp	tus	inhibitor of replication at Ter, DNA-binding protein	Regulatory
2209779	2209780	snp	tus	inhibitor of replication at Ter, DNA-binding protein	Regulatory
2345998	2345999	insertion	ydiP	putative DNA-binding transcriptional regulator	Regulatory
2601352	2601352	deletion	tar	methyl-accepting chemotaxis protein II	Regulatory
2879312	2879313	snp	yegX	putative family 25 glycosyl hydrolase	Others
2900295	2900296	snp	yehH	molybdate metabolism regulator	Regulatory
2943358	2943359	snp	yehX	putative ATP-binding component of a transport system	Others
2946233	2946234	snp	sanA	DUF218 superfamily vancomycin high temperature exclusion protein	Others
3078378	3078379	snp	yfaQ	tandem DUF2300 domain protein, putative host defense protein	Others

bioRxiv preprint doi: https://doi.org/10.64898/2026.06.04.729935; this version posted June 6, 2026. The copyright holder for this preprint (which was not certified by peer review) is the author/funder, who has granted bioRxiv a license to display the preprint in perpetuity. It is made available under a CC-BY 4.0 International license.

3078380	3078380	deletion	yfaQ	tandem DUF2300 domain protein, putative host defense protein	Others
3256833	3256834	snp	fadL	long-chain fatty acid outer membrane transporter	Cell envelope
3367479	3367480	snp	xapA	purine nucleoside phosphorylase 2; nicotinamide 1-beta-D-riboside synthase	Metabolic
3384508	3384509	snp	yffL	CPZ-55 prophage; uncharacterized protein	Regulatory
3398668	3398669	snp	eutE	aldehyde oxidoreductase, ethanolamine utilization protein	Metabolic

Supplementary Table 11. Functional classification of mutations in the Metoclopramide-evolved trajectory Trj2 (folB inhibition).

Start	End	Variant Type	Name	CDS	Gene_type
3808100	3808101	snp	glcD	glycolate oxidase subunit, FAD-linked	Metabolic
3620490	3620490	deletion	aas	fused 2-acylglycerophospho-ethanolamine acyl transferase/acyl-acyl carrier protein synthetase	Metabolic
3513758	3513759	snp	cysI	sulfite reductase, beta subunit, NAD(P)-binding,heme-binding	Others
3507297	3508520	insertion	casA	CRISP RNA (crRNA) containing Cascade antiviral complex protein	Regulatory
3375625	3375626	snp	ypjI	pseudogene, CP4-57 putative prophage remnant;Phage or Prophage Related	Regulatory
3174041	3174042	snp	hyfD	hydrogenase 4, membrane subunit	Cell envelope
2781715	2781896	insertion	yejM	essential inner membrane DUF3413 domain-containing protein; lipid A production and membrane permeability factor	Cell envelope
2579379	2579379	deletion	wcaI	putative glycosyl transferase	Metabolic
2542678	2542679	snp	hisB	histidinol-phosphatase and imidazoleglycerol-phosphate dehydratase	Others

bioRxiv preprint doi: https://doi.org/10.64898/2026.06.04.729935; this version posted June 6, 2026. The copyright holder for this preprint (which was not certified by peer review) is the author/funder, who has granted bioRxiv a license to display the preprint in perpetuity. It is made available under a CC-BY 4.0 International license.

2280352	2281129	insertion	yeaP	diguanylate cyclase	Others
2226964	2226965	snp	ydjY	putative ferrodoxin-like lipoprotein	Cell envelope
1900801	1900801	deletion	ddpX	D-ala-D-ala dipeptidase, Zn-dependent	Efflux and Stress response
1710334	1710334	deletion	fnrS		Others
1362766	1362766	deletion	yceA	putative rhodanese-related sulfurtransferase	Others
1039806	1039807	snp	mntR	Mn(2+)-responsive manganese regulon transcriptional regulator	Regulatory
773308	773309	snp	ybdN	PAPS reductase-like domain protein	Others
459494	459495	snp	yaiO	outer membrane protein	Cell envelope
459493	459494	snp	yaiO	outer membrane protein	Cell envelope
459492	459493	snp	yaiO	outer membrane protein	Cell envelope
441941	441942	snp	lacZ	eta-D-galactosidase	Others
323273	323274	insertion	insI1	IS30 transposase	Transposable element (TE) and Recombination
67920	67920	deletion	folD	folD/ 5,10-methylene-tetrahydrofolate cyclohydrolase	Folate pathway
4419215	4419216	snp	yhhI	putative transposase	Transposable element (TE) and Recombination
4398232	4398233	snp	zntA	zinc, cobalt and lead efflux system	Efflux and Stress response
4355169	4355170	snp	glgB	1,4-alpha-glucan branching enzyme	Efflux and Stress response
4352294	4352295	snp	glgX	glycogen debranching enzyme	Efflux and Stress response
4220149	4220150	snp	gspL	general secretory pathway component	Others
4220148	4220148	deletion	gspL	general secretory pathway component	Others
4041694	4041695	snp	infB	translation initiation factor IF-2	Others
3931048	3931049	snp	ebgA	evolved beta-D-galactosidase, alpha subunit	Metabolic
3914019	3914020	snp	dnaG	DNA primase	Regulatory
3899859	3899860	snp	ygiM	SH3 domain protein	Regulatory

bioRxiv preprint doi: https://doi.org/10.64898/2026.06.04.729935; this version posted June 6, 2026. The copyright holder for this preprint (which was not certified by peer review) is the author/funder, who has granted bioRxiv a license to display the preprint in perpetuity. It is made available under a CC-BY 4.0 International license.

Supplementary Table 12. Functional classification of mutations in the Metoclopramide-evolved trajectory Trj3 (folB inhibition).

Start	End	Variant Type	Gene Name	CDS	Gene_type
3808100	3808101	snp	glcD	glycolate oxidase subunit, FAD-linked	Metabolic
3620490	3620490	deletion	aas	fused 2-acylglycerophospho-ethanolamine acyl transferase/acyl-acyl carrier protein synthetase	Metabolic
3513758	3513759	snp	cysI	sulfite reductase, beta subunit, NAD(P)-binding,heme-binding	Metabolic
3507297	3508520	insertion	recN	recombination and repair protein	Transposable element (TE) and Recombination
3375625	3375626	snp	ypjI	pseudogene, CP4-57 putative prophage remnant	Regulatory
3174041	3174042	snp	hyfD	hydrogenase 4, membrane subunit	Cell envelope
2781715	2781896	insertion	yejM	essential inner membrane DUF3413 domain-containing protein; lipid A ion and membrane permeability factor	Cell envelope
2579379	2579379	deletion	wcaI	putative colanic biosynthesis glycosyl transferase	Metabolic
2542678	2542679	snp	hisB	histidinol-phosphatase and imidazoleglycerol-phosphate dehydratase	Metabolic
2280352	2281129	insertion	yeaP	diguanylate cyclase	Others
2226964	2226965	snp	ydjY	putative ferrodoxin-like lipoprotein	Cell envelope
1900801	1900801	deletion	ddpX	D-ala-D-ala dipeptidase, Zn-dependent	Metabolic
1710334	1710334	deletion	dbpA	ATP-dependent RNA helicase, specific for 23S rRNA	Regulatory
1362766	1362766	deletion	yceA	putative rhodanese-related sulfurtransferase	Metabolic
1039806	1039807	snp	mntR	Mn(2+)-responsive manganese regulon transcriptional regulator	Regulatory
773308	773309	snp	ybdM	Spo0J family protein, ParB-like nuclease domain	Others
459494	459495	snp	yaiO	outer membrane protein	Cell envelope
459493	459494	snp	yaiO	outer membrane protein	Cell envelope
459492	459493	snp	yaiO	outer membrane protein	Cell envelope
434952	434953	snp	yaiO	outer membrane protein	Cell envelope

bioRxiv preprint doi: https://doi.org/10.64898/2026.06.04.729935; this version posted June 6, 2026. The copyright holder for this preprint (which was not certified by peer review) is the author/funder, who has granted bioRxiv a license to display the preprint in perpetuity. It is made available under a CC-BY 4.0 International license.

323273	323274	insertion	insI1	IS30 transposase	Transposable element (TE) and Recombination
67920	67920	deletion	lptD	LPS assembly OM complex LptDE, beta-barrel component	Others
4419215	4419216	snp	prlC	oligopeptidase A	Metabolic
4398232	4398233	snp	zntA	zinc, cobalt and lead efflux system	Efflux and Stress response
4355169	4355170	snp	glgX	glycogen debranching enzyme	Efflux and Stress response
4352294	4352295	snp	glgX	glycogen debranching enzyme	Efflux and Stress response
4220149	4220150	snp	gspL	general secretory pathway component, cryptic	Others
4220148	4220148	deletion	gspL	general secretory pathway component, cryptic	Others
4041694	4041695	snp	infB	translation initiation factor IF-2	Metabolic
3931048	3931049	snp	ebgA	evolved beta-D-galactosidase, alpha subunit	Metabolic
3914019	3914020	snp	ebgA	evolved beta-D-galactosidase, alpha subunit	Metabolic
3899859	3899860	snp	ygiF	inorganic triphosphatase	Others

Supplementary Table 13. Gene family-level organization of mutations across Metoclopramide-evolved trajectories

Gene Family	Trj1	Trj2	Trj3	Similarity Pattern
glc*	glcB	glcD	glcD	Same metabolic family
cys*	cysC	cysI	cysI	Sulfur assimilation family
his*	hisS	hisB	hisB	Histidine pathway family
yej*	–	yejM	yejM	Same gene
ydd/ydj*	–	ydjY	ydjY	Same gene
glg*	–	glgB, glgX	glgX	Glycogen metabolism family
gsp*	–	gspL	gspL	Secretion system family

bioRxiv preprint doi: https://doi.org/10.64898/2026.06.04.729935; this version posted June 6, 2026. The copyright holder for this preprint (which was not certified by peer review) is the author/funder, who has granted bioRxiv a license to display the preprint in perpetuity. It is made available under a CC-BY 4.0 International license.

ins*	–	insI1	insI1	Transposable element family
mntR / metal regulation family	–	mntR	mntR	Same gene
ddp*	–	ddpX	ddpX	Dipeptide metabolism family
yaiO / envelope gene	–	yaiO	yaiO	Same gene
wca*	–	wcaI	wcaI	Capsule biosynthesis family
